# Dietary Protein and Muscle in Aging People: The Potential Role of the Gut Microbiome

**DOI:** 10.3390/nu10070929

**Published:** 2018-07-20

**Authors:** Mary Ni Lochlainn, Ruth C. E. Bowyer, Claire J. Steves

**Affiliations:** 1 The Department of Twin Research, Kings College London, 3-4th Floor South Wing Block D, St Thomas’ Hospital, Westminster Bridge Road, London SE1 7EH, UK; ruth.c.bowyer@kcl.ac.uk (R.C.E.B.); claire.j.steves@kcl.ac.uk (C.J.S.); 2 Clinical Age Research Unit, Kings College Hospital Foundation Trust, London SE5 9RS, UK

**Keywords:** protein, skeletal muscle, sarcopenia, gut microbiome, metabolome, diet, supplementation

## Abstract

Muscle mass, strength, and physical function are known to decline with age. This is associated with the development of geriatric syndromes including sarcopenia and frailty. Dietary protein is essential for skeletal muscle function. Resistance exercise appears to be the most beneficial form of physical activity for preserving skeletal muscle and a synergistic effect has been noted when this is combined with dietary protein. However, older adults have shown evidence of anabolic resistance, where greater amounts of protein are required to stimulate muscle protein synthesis, and response is variable. Thus, the recommended daily amount of protein is greater for older people. The aetiologies and mechanisms responsible for anabolic resistance are not fully understood. The gut microbiota is implicated in many of the postulated mechanisms for anabolic resistance, either directly or indirectly. The gut microbiota change with age, and are influenced by dietary protein. Research also implies a role for the gut microbiome in skeletal muscle function. This leads to the hypothesis that the gut microbiome might modulate individual response to protein in the diet. We summarise the existing evidence for the role of the gut microbiota in anabolic resistance and skeletal muscle in aging people, and introduce the metabolome as a tool to probe this relationship in the future.

## 1. Introduction

**Figure 1:**
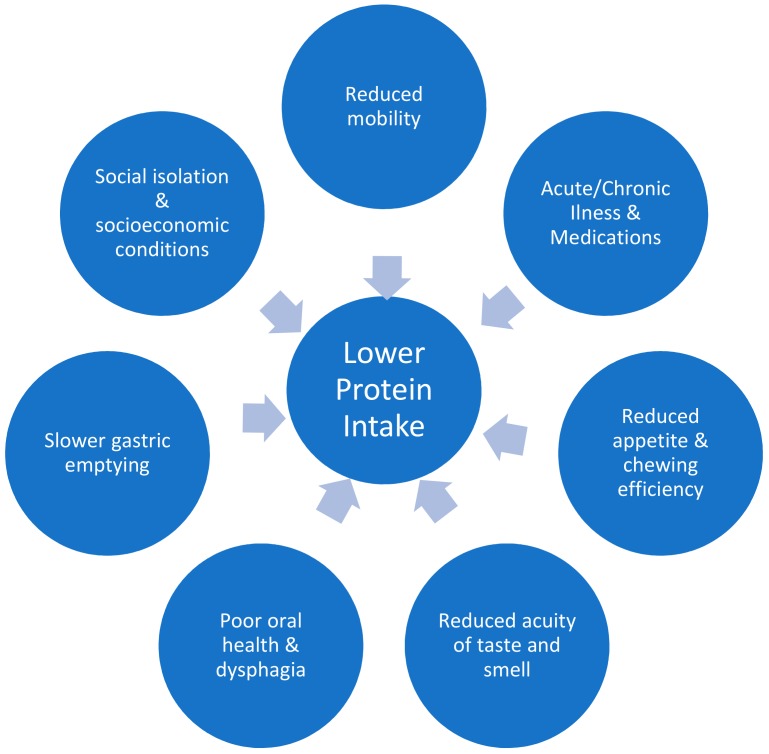
Factors leading to lower protein intake in older adults.

Skeletal muscle has several important functions beyond locomotion, including insulin-stimulated glucose uptake, influence on bone density via mechanical force on bones, and whole-body protein metabolism [1]. Age associated loss of muscle mass starts as early as age thirty, and is a gradual process [1]. Older people lose more skeletal muscle with bedrest and show an attenuated response to retraining after immobilisation, in comparison to younger individuals [2,3,4]. Sarcopenia is a geriatric syndrome defined as the age-related loss of skeletal mass and function, quantified by objective measures of muscle mass, strength, and physical function [5]. One major risk factor for the development of sarcopenia is protein-energy malnutrition [6]. A number of factors can lead to reduced protein intake in older age, as summarised in Figure 1 [7,8,9,10,11,12,13,14,15,16,17,18]. Patients with sarcopenia are often frail (vulnerable to minor stressors) and the two concepts (frailty and sarcopenia) share an increased risk of adverse outcomes [19]. As life expectancy worldwide has more than doubled over the past two centuries, the importance of understanding and optimising muscle function in older age is paramount.

Three large observational studies have supported an association between protein intake and muscle strength and mass [20,21,22], but multiple trials carried out in healthy, replete, older adults, without an exercise intervention, have been negative [23,24,25]. In those with suboptimal protein intake, the most promising results are for specific essential amino acids, particularly leucine, but also its metabolite β-hydroxy β-methylbutyric acid (HMB) [25,26,27,28,29]. Supplementation with these more targeted regulators of muscle protein synthesis (MPS) may be most effective for overcoming anabolic resistance in this cohort, especially if combined with exercise, a potent stimulator of anabolic response in muscle at all ages [28,30,31,32]. Anabolic resistance refers to the phenomenon whereby older adults require a higher dose of protein to achieve the same response in MPS as a younger adult [1]. The aetiologies and mechanisms for this are not understood, but we propose that the gut microbiome may be implicated in one or many of those suggested in the literature.

The gut microbiome is composed of bacteria, archaea, viruses, and eukaryotic microbes that reside in the gut. Its role in maintaining a healthy physiology and contributing to disease is a rapidly evolving field of enquiry. The gut microbiome has a collective genome size that may be as much as 150-fold that of the human host [33], and it has been argued that the metabolic capacity of microbiota merits its consideration as an organ of the human body in its own right, with its own intrinsic functions and metabolic needs [34]. With age and frailty in particular, the resilience of the gut microbiome is reduced, as it becomes more vulnerable to medications, disease, and changes in lifestyle, with changed species richness and increased inter-individual variability [35,36,37]. The potential of the gut microbiota to alter physiology has been shown by landmark animal studies assessing faecal transplant, which have demonstrated body composition changes in the recipient reflective of the phenotype of the donor [38]. This highlights the role of microbiota in characterising metabolic phenotypes, which we are only now beginning to understand.

Ageing is associated with chronic inflammation [39], often referred to as ‘inflammaging’. Here we suggest that this ‘inflammaging’, in combination with altered gut microbiome composition and/or diversity [40], leads to changes in protein metabolism, absorption and availability; ultimately contributing to anabolic resistance and therefore to reduced MPS and the development of sarcopenia. Proposed interventions such as protein supplementation, probiotics or faecal transplants should address this rationale. This review summarises the available literature on anabolic resistance in older adults, with a particular focus on the role of the gut microbiome and its metabolome.

## 2. Anabolic Resistance

**Figure 2:**
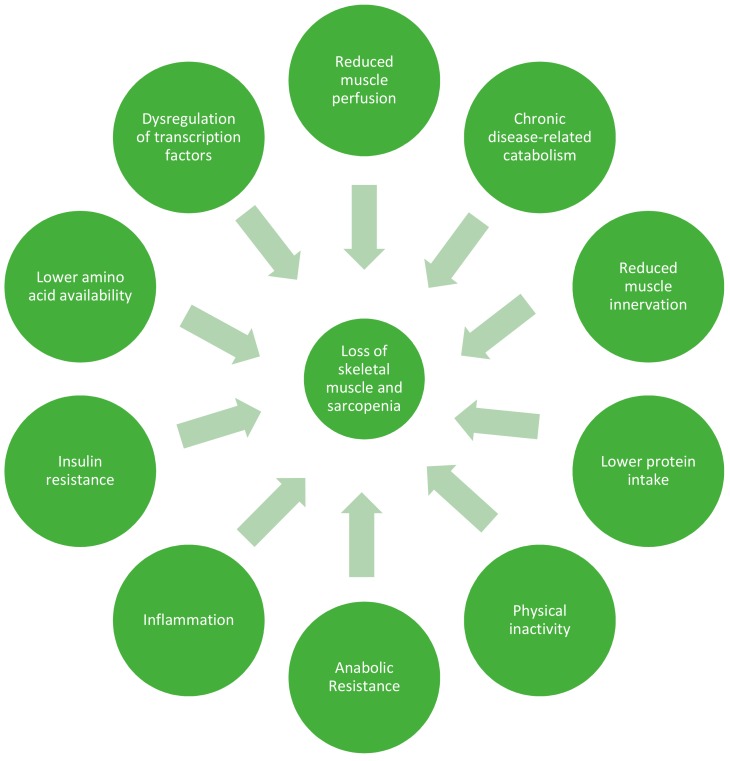
Factors leading to loss of skeletal muscle and sarcopenia in older adults.

Skeletal muscle mass is regulated by the processes of muscle protein synthesis and breakdown (MPS and MPB). MPS rates are largely controlled by responsiveness to anabolic stimuli, such as consumption of food, and physical activity. Catabolic stressors include illness, physical inactivity, and inflammation, of which the older population tend to have higher rates (Figure 2). Ageing does not seem to influence MPB to the same degree as MPS, and so much of the focus of the aging literature is on MPS [27,41,42,43]. Older adults have shown evidence of ‘anabolic resistance’, whereby a higher dose of protein is required to achieve the same MPS response as a younger person [1,28,39,40,44]. While this concept has been questioned, especially in the context of healthy older adults [45], it is now considered consensus that a higher recommended daily amount of 1–1.3 g/kg/day should be consumed by older people to offset catabolic conditions [1,46,47,48,49]. In the context of illness or injury, older adults may require as much as 2 g/kg/day, as recommended by the PROT-AGE Study Group [50].

The aetiology of anabolic resistance is complex, involving aging physiology, accumulation of chronic disease, and changes in physical inactivity (see Table 1). The multiple mechanisms postulated involve impairments at most levels of protein metabolism (see Table 2). There may also be sex-differences in anabolic resistance [51,52,53,54], which has received little attention in the literature.

## 3. The Role of the Gut Microbiome

The composition of the human gut microbiome is dependent on, amongst other things, age, diet, health, and geographical location, with significant individual variability [94,95]. It is dynamic across the lifespan, changing rapidly between birth and early childhood, and then becoming more stable [36]. In older life, however, research shows that the propensity for compositional change accelerates once again [36,96,97]. Multiple cross-sectional studies have found associations between gut microbiome composition and frailty [98,99,100], while the ELDERMET study showed significant loss of diversity amongst people in a care-home setting versus community dwellers [95]. Among older hospitalised patients, polypharmacy has been associated with gut microbiota dysbiosis [99]. It is well established that antibiotics cause significant changes in microbiota composition [101], and older adults tend to have more frequent antibiotic therapy.

Age-related chronic inflammation (‘inflammaging’), is implicated in the development of sarcopenia [102,103]. Changes in the gut microbiota have been suggested to contribute to inflammaging [37,103,104,105]. A recent animal study showed that transferring gut microbes of young killifish to older ones ameliorates ageing conditions, and extends the lifespan of the older fish [106]. Notably, the transplanted older fish also displayed increased ‘spontaneous exploratory behaviour’ [106], essentially physical activity. Few studies to date have had the ability to delve into the operational capacity and functional readout of the gut microbiome in relation to aging, but this is likely to shed more light on possible mechanisms of the interaction between dietary intake and host utilisation of protein in skeletal muscle.

### 3.1. Gut Microbiota and Skeletal Muscle

The influence of the gut microbiome in metabolic health has been one of the primary focuses of research in this area thus far, particularly in the context of obesity and insulin resistance [107]. Studies have used faecal transplants in germ-free mice to demonstrate changes in body fat, insulin resistance and glucose tolerance [108], highlighting the key role of the microbiome in these metabolic pathways. Considering the role of skeletal muscle in glucose metabolism, animal studies have investigated the relationship between gut microbiota and skeletal muscle metabolism. For example, skeletal muscle from colonised versus germ free mice appears to have altered metabolic efficiency, with higher levels of the enzyme adenosine monophosphate (AMP)-activated protein kinase, a central regulator of metabolism at both a cellular and organismal level, found in the skeletal muscle of germ-free mice [109]. CD-14 mutant mice, who lack an endotoxin receptor on their innate immune cells, have increased levels of circulating lipopolysaccharide (LPS), and this LPS was found to induce skeletal muscle inflammation, as well as insulin resistance [36]. This is important because the healthy gut microbiome is considered to contribute to gut barrier function (Section 3.3 below), providing gut enterocytes with essential nutrition [110] and reducing LPS levels in the blood. Lastly, Yan et al. (2016) carried out a study in which gut microbiota was transferred from obese pigs to germ free mice [111]. Fibre characteristics and the metabolic profile of the skeletal muscle were replicated in the recipients [111], again implicating the gut microbiome in skeletal muscle composition and metabolism. Some of the fibre changes noted were similar to those seen in aging skeletal muscle (e.g., increased proportion of slower contracting fibres). This raises the possibility that faecal microbial transplantation could be used as a means to transmit muscle fibre characteristics between humans, perhaps even from young to old, as a means of improving skeletal muscle function.

Gut microbiota modulation in animal models has also produced preliminary supportive data for effect on skeletal muscle. This includes lower intestinal permeability and lower plasma LPS and cytokines noted in prebiotic-treated mice [112], reduced expression of muscle atrophy markers in mice models of leukaemia supplemented with a *Lactobacillus* species [113], and increased muscle mass and function (measured by grip strength and swim time) in healthy mice supplemented with *L. plantarum* [114]. These studies and others [115,116], suggest that targeting the gut microbiota may be used as a tool to modulate muscle mass.

In terms of human data, two probiotic trials have shown improvements in athletic performance amongst elite athletes. A small, four week trial of probiotic capsules in male runners reported increased run time to fatigue in the probiotic group [117], while a trial of probiotic yoghurt in teenage female endurance swimmers reported improved aerobic performance, measured by maximal oxygen consumption (VO2 max) [118]. Dietary standardisation was carried out in the male runner study, however in the swimmer study participants continued their regular diet which may have confounded results. These studies build on evidence from observational studies for an association between exercise and gut microbiota [119,120,121,122,123,124]. Clark et al. (2014) compared the gut microbial diversity of professional male athletes to healthy controls and reported significantly higher diversity amongst the athletes [125]. Furthermore, moderate exercise has been shown to increase intestinal mobility [126], which is known to affect gut microbiota [127,128]. These changes in gut health with exercise implicate skeletal muscle as a potential regulator of gut microbiota composition and suggest a bi-directional relationship between skeletal muscle and the gut microbiome.

Amongst older adults, a single randomised controlled trial has explored the effect of modulating the gut microbiota on muscle function and frailty. Here, 60 older adults received a prebiotic (F-GOS) or placebo for 13 weeks. While the study remains to be replicated, promisingly, both exhaustion and handgrip strength were significantly improved in the treatment arm [129], highlighting the potential role for the gut microbiome in future interventions. The science of pre- and probiotic use is in its infancy, as are studies of faecal transplantation, with much scope for further investigation of these therapeutic options.

### 3.2. Gut Microbiota and Dietary Protein

The digestive system consists of a complex interaction between digestive secretions, intestinal conditions, and the gut microbiome. Nutrients, especially dietary proteins, provide energy sources for the host, as well as substrates for the gut microbiota [130]. A significant proportion of undigested peptides and proteins can reach the colon, and this is increased in the context of a high protein diet [131]. Consumption of proteins with high digestibility, or a low protein diet, results in less protein reaching the colon, limiting the amount available for protein-fermenting bacteria [130]. Furthermore, changes in the gut microbiota can impact the bioavailability of dietary amino acids [104,132].

Studies carried out in mice, rats, and hamsters have shown higher microbial diversity in those fed soy protein versus animal protein [133,134] and increased abundance of *Bacteroidales* family S24-7 in those fed soy protein versus other diets [79]. Li et al. (2017) assessed high protein, low carbohydrate diets in dogs and found decreased *Bacteroidetes* to *Firmicutes* ratio, increased *Bacteroides* to *Prevotella* ratio and increased abundance of *Clostridium hiranonis*, *Clostridium perfringens*, and *Ruminococcus gnavus*, the latter of which has been proposed to have beneficial effects in the human gut [135].

It has been reported that protein consumption is correlated positively with gut microbiota diversity [136]. This is based on studies carried out on healthy volunteers [137], elite athletes [125], and obese/overweight individuals [138]. The source of protein appears influential, with plant protein associated with more *Bifidofacterium*, *Lactobacillus*, *Roseburia*, *Eubacterium rectale*, and *Ruminococcus bromii*; and less *Bacteroides* and *Clostridium perfringens* [136,137]. Meanwhile animal protein was associated with higher levels of *Bacteroides*, *Alistipes*, *Bilophila* and *Ruminococcus*, and lower levels of *Bifidobacterium* [136,137]. High levels of *Bacteroides* have also been reported with Western diets, which are high in protein and animal fat [33], although it has been suggested that differences in fat content, rather than protein, is the major influencing factor here [139]. Significant associations have been reported between increased levels of faecal short chain fatty acids (SCFAs), *Prevotella* and some *Firmicutes*, with consumption of a Mediterranean diet [35,140], which is typically lower in protein than animal-based diets, although may contain high levels of plant-source protein. Dietary fibre is an important factor in gut microbiome diversity and composition and it is important to note that most plant sources of protein are also high in fibre, whereas animal source protein are not. This is likely to be an influential factor in the findings of these studies.

The gut microbiomes of critically ill patients on average display enrichment of virulent pathogens, and loss of health-promoting microbes [141]. Protein supplementation has shown some benefits for muscle parameters in this population [142,143], but whether this effect is modulated by the gut microbiome remains to be tested. Evidently dietary protein has a significant effect on gut microbiota composition and vice versa, however more research is needed to further characterise this relationship. It is notable that almost exclusively, studies to date have focused on composition of the microbiota rather than functional capacity of the microbiome. Investigation into the differences in microbial genes involved in protein metabolism between individuals differing in anabolic response to protein could lead to the engineering of new probiotics with specific capacity to influence MPS.

### 3.3. Gut Microbiota and Anabolic Resistance

**Figure 3:**
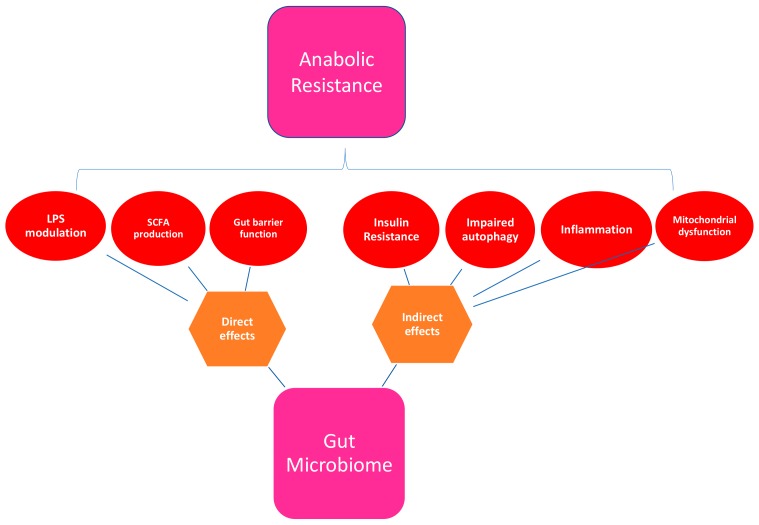
Mechanisms by which the gut microbiome may influence anabolic resistance. LPS: Lipopolysaccharide; SCFA: Short chain fatty acids.

A healthy gut microbiome plays a role in many of the physiological processes implicated in the various mechanisms proposed for the development of anabolic resistance (see Table 2 and Figure 3). These include suppression of chronic inflammation, prevention of insulin resistance, modulation of host gene expression, enhancement of antioxidant activity, and maintenance of gut barrier function [35,104].

Inflammation has been proposed as a contributing factor to anabolic resistance in aging, and indeed inflammaging has been suggested as a major aetiological factor in the development of sarcopenia. Biagi et al. (2010) studied age-related differences in both the gut microbiota and the inflammatory status among different stages of the whole adult life, including centenarians, and reported dysbiosis in the older population, which correlated with increased inflammatory status, as determined by peripheral blood inflammatory markers [37].

Work in animal models has shown evidence of increased intestinal permeability in association with age-associated microbial dysbiosis [36,104,144]. This can facilitate translocation of microbial byproducts into the circulation, including endotoxins, and may influence a number of the mechanisms listed in Table 2, such as protein digestion and absorption. It has been suggested that pathogenic drivers of inflammation and muscle atrophy may enter the system via this process [132]. Within humans, a randomised controlled trial of probiotic use in athletic men reported reduced zonulin in faeces, a surrogate marker of enhanced gut permeability [145], suggesting that modulation of the gut microbiota can affect the gut’s barrier function.

Older adults tend to have reduced intestinal motility, which may unfavourably affect the utilisation of dietary protein by the gut [104]. Indeed it has been reported that the proteolytic potential of the gut microbiota appeared to be enhanced in older age [146], and may therefore contribute to anabolic resistance to ingested protein. There is also some evidence that probiotics may improve amino acid absorption from protein [147,148], which adds weight to the suggestion that targeting the gut microbiota may ameliorate anabolic resistance in older adults. Production of SCFAs by the gut microbiota has been associated with anabolism itself [110] and depletion of taxa producing SCFAs may promote anabolic resistance [149]. Of note, an age-related reduction of the abundance of genes in pathways that are involved in SCFA production has been reported [146]. SCFAs are mainly produced by the fermentation of dietary fibre, so the fibre content of dietary protein sources is likely too, to influence protein metabolism.

Treatment with butyrate (a SCFA), which is associated with *Bifidobacterium*, was found to be protective of muscle atrophy in mice [116]. Notably, studies showing correlation between frailty and gut microbiota composition have also reported dysbiotic shifts in higher functioning older adults towards a greater abundance of butyrate-producing bacteria such as *Faecalibacterium prausnitzii* [95,150], which suggests these microbes may have a positive role in protection against muscle loss and frailty. Butyrate also has a role in intestinal barrier function [151], and therefore may be implicated in intestinal permeability. Notably, a randomised controlled trial of symbiotic (a combination of pre- and probiotic) use in older people noted an increase in butyrate production in those given the synbiotic [152].

Mitochondrial dysfunction and impaired autophagy have both been suggested as possible mechanisms for anabolic resistance (see Table 2). Interestingly, they have also been implicated in animal models of aging [153] and in the development of sarcopenia and cachexia [154,155]. A recent paper has postulated that dysfunctional mitochondria may represent a key link between chronic inflammation and age-related muscle loss, and that dysbiosis of the gut microbiota may be a key mediator in this gut-muscle crosstalk [104].

Evidently, there are multiple mechanisms by which the gut microbiome may influence anabolic resistance in older adults (see Figure 3), and it is likely to be a complex interaction between a number of, if not all, of these postulated processes. The hypothesis that the dysbiotic gut plays a role in the loss of skeletal muscle and response to protein is yet to be tested. If supported, the gut microbiota could represent a target for interventions aiming to overcome anabolic resistance, to maintain muscle mass and strength in older adults, with the aim of ultimately preventing the development of sarcopenia and/or frailty.

### 3.4. The Metabolome

Studies use multiple ways of estimating dietary protein intake. The validity and reliability of these dietary measures has usually been verified in younger populations and may not be relevant to older people. Indeed reduced reliability coefficients of the Food Frequency Questionnaire have been reported with increasing age [156]. In order to overcome this, researchers have sought objective estimates of dietary intakes. Protein is the major nitrogen-containing substance in the body, and therefore urinary excretion of nitrogen is used as a marker of protein loss [23,40]. Urinary [31,131] and blood urea concentration [131], and urinary HMB levels [157] have also been used with the aim of objectively verifying compliance. These methods are not without limitations, as they may not consider subtle changes with protein metabolism that occur with age, such as increased splanchnic uptake [50]. The amount of fermentation metabolites detectable in the urine depends on the digestibility of the protein [130], so this too needs to be considered. Another way to study gut microbiota composition is altered fermentation products. Promisingly, the faecal metabolome has been shown to be largely reflective of gut microbial composition [158]. Trials using ^1^H-nuclear magnetic resonance (NMR) technology have shown a shift in bacterial metabolism with different metabolite profiles according to the source of protein [131]. A growing number of studies are using ^1^H-NMR technology to assess faecal, urinary, and plasma metabolomes as measures of metabolic health (e.g., [159]). More research is needed into the use of the metabolome in the context of dietary protein intake, and the significance of metabolome changes for skeletal muscle mass and function.

## 4. Discussion

As the world’s population ages, it has become imperative to gain more understanding of the aging process. Declines in muscle mass and function with age have significant associated morbidity and mortality, and the prevalence of both sarcopenia and frailty is increasing. The care of older people is complex, and a multitude of factors influence lower protein intake and loss of skeletal muscle with age (see Figure 1). Studies show that supplementing protein, particularly in combination with resistance exercise, is beneficial for aging muscle. However, trials have had conflicting results. Perhaps a more personalised approach is warranted? Attempting to answer this question is a large randomised controlled trial, currently being carried out, on personalised dietary recommendations as part of a multi-component intervention in the management of sarcopenia [160].

Anabolic resistance is likely to result from cumulative declines across multiple physiological systems, with effects on both MPS and MPB, a dynamic interaction of multiple factors (see Figure 2). Current thinking must not be limited to one or two mechanisms but focus on anabolic resistance as a complex and multidimensional construct. The aetiologies and mechanisms involved are not understood and may be different for each aging individual, again suggesting a potential need for personalised medicine within this population to guide future interventions. The potential role of the gut microbiota in a substantial number of postulated mechanisms for anabolic resistance warrants further investigation (Figure 3). Targeting the gut microbiota to overcome anabolic resistance holds promise in maximising responses in participants who can undertake exercise programs, but where resources and time limit such programs. Moreover, the potential ability to influence skeletal muscle function via gut microbiota in the context of those who cannot feasibly carry out vigorous exercise programs is also an attractive idea.

Few human studies have evaluated the effects of the gut microbiome on dietary protein metabolism, and the ensuing metabolome or vice versa. Studies addressing the role of the gut microbiota in skeletal muscle function are also limited in number. Animal studies have shown promise, and one human trial in older adults showed positive improvements in muscle function with prebiotic gut microbiome modulation [129]. Furthermore, in light of difficulties in accurately capturing an individual’s dietary intake from questionnaire data [161], the use of the metabolome may represent an objective and reliable way of assessing compliance with dietary interventions going forward [162], and provide a functional readout for the gut microbiome.

To date there is some supporting evidence for a hypothesis that the gut microbiome may influence the health of skeletal muscle and vice versa [35,36,104], however this remains to be formally tested. In particular, processes such as muscle metabolism and inflammation may be susceptible to modulation. Research is needed to establish whether deleterious changes in the gut microbiome contribute to skeletal muscle loss in the context of acute or chronic illness, or changes detected in apparently healthy aging. The plasticity and diversity of the gut microbiome and its metabolome, represent exciting prospects to individualise the response of skeletal muscle in older adults to dietary protein.

## Figures and Tables

**Table 1:** Factors influencing anabolic resistance.

Anabolic Resistance Aetiology	References
Declining activity levels	[1,6,55,56,57]
Protracted disuse events	[6,58,59,60,61]
Chronic inflammation	[31,41,56,62,63]
Insulin resistance	[1,27,41,62,64,65]
Higher circulating oxidative and inflammatory stressors	[1,27,56]
Obesity	[62,66]
Reduced oestrogen/testosterone	[1,67]
Increased production of catabolic hormones such as cortisol	[27]
Alcohol	[68]
Smoking	[1]
Poor vitamin D status	[56]
Reduced food intake	[56]
Metabolic acidosis	[1]
More chronic & acute disease in older adults (increased catabolic conditions)	[50]

**Table 2:** Molecular mechanisms implicated in anabolic resistance.

Anabolic Resistance Mechanisms	References
Differences in gene expression of proteins involved in MPS	[69,70,71,72,73]
Dysregulation of key signalling proteins in the mTOR pathway	[1,41,70,71,74,75]
Decreased phosphorylation of mTORC1	[41,74,76,77,78,79]
Impaired transport of amino acids into muscle/peripheral tissues	[56,75,80,81]
Diminished mRNA translational signalling	[74,78,82,83]
Inflammation (raised TNFα/IL-6/hs-CRP/NFkB)	[1,41,74,84,85]
Decreased phosphorylation of transcription factors (e.g., p70S6K, S6K1)	[41,74,75,82]
Dysregulation of nutritive blood flow to skeletal muscle	[56,65,86]
Attenuated protein digestion & absorption	[56,87,88,89]
Mitochondrial dysfunction	[1,35,72]
Autophagy/mitophagy dysfunction	[1,72]
Denervation of muscle fibres	[56,90]
Higher splanchnic extraction of protein	[50,88]
Lipid-induced muscle insulin resistance	[35,91]
Increased AMPKα phosphorylation (leads to increased MPB)	[70]
Increased cortisol generation within muscle by 11bHSD1	[92]
Loss of skeletal muscle stem cells	[93]
